# Just-in-time deep learning for real-time X-ray computed tomography

**DOI:** 10.1038/s41598-023-46028-9

**Published:** 2023-11-16

**Authors:** Adriaan Graas, Sophia Bethany Coban, K. Joost Batenburg, Felix Lucka

**Affiliations:** 1 Computational Imaging, CWI, 1098 XG Amsterdam, The Netherlands; 2 Leiden Institute of Advanced Computer Science, Leiden University, Niels Bohrweg 1, 2333 CA Leiden, The Netherlands; 3 Frazer-Nash Consultancy, Leatherhead, KT22 7NL Surrey UK

**Keywords:** Applied mathematics, Computational science, Imaging techniques

## Abstract

Real-time X-ray tomography pipelines, such as implemented by RECAST3D, compute and visualize tomographic reconstructions in milliseconds, and enable the observation of dynamic experiments in synchrotron beamlines and laboratory scanners. For extending real-time reconstruction by image processing and analysis components, Deep Neural Networks (DNNs) are a promising technology, due to their strong performance and much faster run-times compared to conventional algorithms. DNNs may prevent experiment repetition by simplifying real-time steering and optimization of the ongoing experiment. The main challenge of integrating DNNs into real-time tomography pipelines, however, is that they need to learn their task from representative data *before* the start of the experiment. In scientific environments, such training data may not exist, and other uncertain and variable factors, such as the set-up configuration, reconstruction parameters, or user interaction, cannot easily be anticipated beforehand, either. To overcome these problems, we developed *just-in-time learning*, an online DNN training strategy that takes advantage of the spatio-temporal continuity of consecutive reconstructions in the tomographic pipeline. This allows training and deploying comparatively small DNNs during the experiment. We provide software implementations, and study the feasibility and challenges of the approach by training the self-supervised Noise2Inverse denoising task with X-ray data replayed from real-world dynamic experiments.

## Introduction

Computed Tomography (CT) is a powerful imaging technique to reveal the interior of objects using X-rays, with applications in health care, industry, life sciences, physics^1^, material sciences^2^, as well as many other fields^3^. At synchrotron light source facilities and X-ray microscopy laboratories, time-resolved tomography allows the reconstruction of dynamically evolving processes^4^. In these environments, experimental data is collected by an imaging scientist, but reconstruction is often postponed to a later stage. As a result, the domain expert has little control or feedback over the imaging process^5,6^, and an experiment may need to be repeated after reconstructions have been inspected. With recent advances in hardware, such as GPUs (*Graphical Processing Units*) and CMOS (*Complementary Metal Oxide Semiconductor*) detector technology, several synchrotrons and laboratories have now developed *real-time* tomographic pipelines. Next to streamlining data acquisition and preprocessing steps, these pipelines achieve reconstructions within milliseconds^5,7–9^. Live observation of the experiment helps image scientists and domain experts to optimize acquisition settings, and therefore save valuable time and storage^4,6,10^.

**Figure 1:**
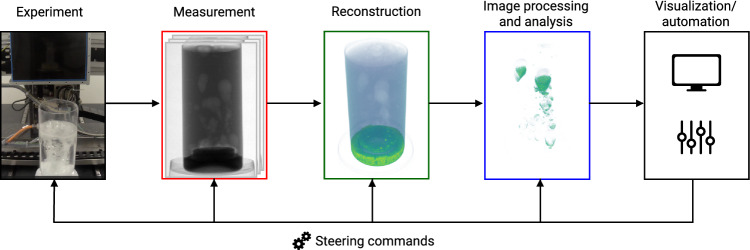
In real-time tomographic pipelines, measurement acquisition, image reconstruction, and image processing or analysis, are subsequent computational steps that can be executed concurrently during an experiment. User feedback or DNN automation can be used to steer the experiment. The illustration shows a dissolving-tablet experiment at the FleX-ray laboratory^5^, and is further detailed in the Experimental set-up section.

While real-time reconstruction provides a valuable insight into the imaged object through the spatial distribution of X-ray attenuation, *image processing and analysis* tasks are often necessary to improve or evaluate the experimental outcome. Algorithms for these tasks can perform image enhancements, such as noise and artefact removal, and show whether or not the reconstructed object features are of sufficient quality. In more complex cases, they may extract semantic information, e.g., through object classification, counting of object instances, or image segmentation. This helps the domain expert in the evaluation of the experiment. However, many image analysis tasks take too much time when computed with traditional algorithms, and integration in tomographic pipelines has therefore not yet been possible. With the advent of Deep Learning, this has changed. A new class of algorithms called *Deep Neural Networks* (DNNs), in particular those using convolutional layers (CNNs), shows remarkable results for a wide range of image tasks, such as motion estimation and image classification^11^. Because DNNs can operate on the timescales required by real-time tomography, they are a promising next component in the tomographic pipeline, which we illustrate in Fig. 1. In the nearby future, DNNs may be able to close the experimentation loop, by providing automated feedback and experimental control^8^. For example, a DNN could automatically identify a region of interest, and adapt the scanning geometry to zoom into it.

In practice, applying DNNs in real-time tomographic pipelines is not straightforward. The success of DNNs is generally attributed to *training*, i.e., the optimization of algorithmic hyperparameters based on data. This process takes place before *inference*, i.e., the application of a network to unseen data. The real-time setting makes training challenging for two main reasons. The first concerns the acquisition of training data. Image reconstructions—the inputs to a neural network—depend on the object to be imaged, the set-up and noise levels, and the reconstruction algorithm. Experiments are, furthermore, often not repeatable, and the effect of user interaction with the experiment cannot easily be anticipated as well. Generating training data of sufficient quality and quantity from previous experiments may therefore be difficult, or relevant training data may simply not be available. The second reason is a lack of training time. Beamline time at synchrotron facilities is scarce and expensive, making schedules too tight to train a DNN in between experiments.

We propose to overcome these challenges by taking advantage of the spatio-temporal continuity of samples from a tomographic pipeline. Unlike typical data sets used to train CNNs, image samples from the pipeline are the result of continuous processes, e.g., the dynamics of the experiment, the mechanics of the set-up, or changes in the reconstruction parameters. Data samples are therefore not i.i.d. samples, but rather possess a high degree of spatio-temporal continuity. This opens the opportunity to train small neural networks that adapt to the data during the experiment, while simultaneously being used for inference on incoming reconstructions. Since patterns in the images are likely to reappear in the following seconds to minutes, we say the network is trained *just-in-time* for inference. This offers the sought flexibility towards changing and unpredictable reconstructions, and can be fully automated, once integrated in a pipeline. The approach is limited to network architectures that can be trained quickly, and is only applicable to learning tasks that do not require external data. However, as the goal is to perform well on the current image from the pipeline, only a small data set is required for training. To the best of our knowledge, we are the first to propose and investigate such an approach for CNNs.

**Figure 2:**
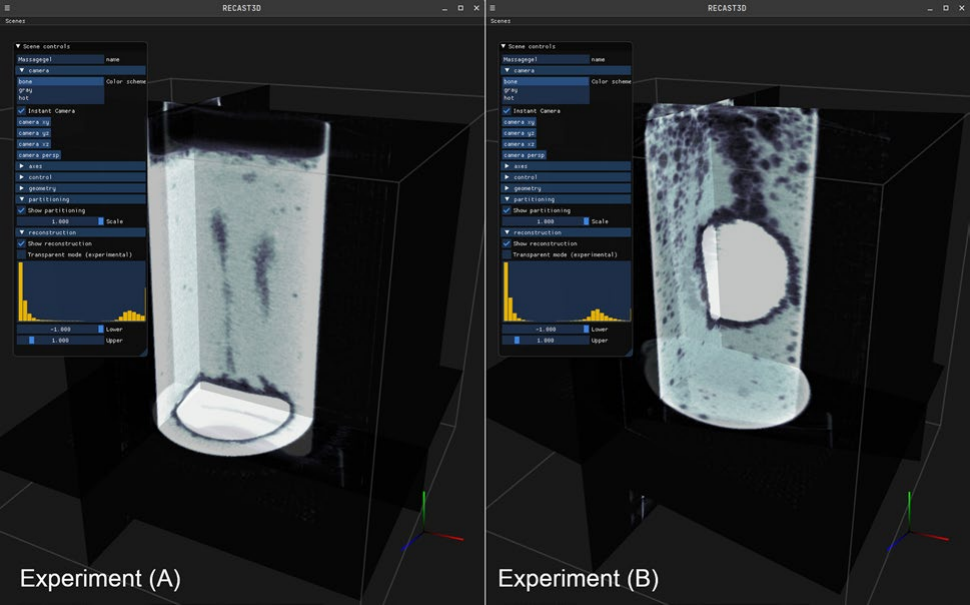
Illustration of the RECAST3D user interface (https://github.com/cicwi/RECAST3D, release v1.1.0). Left: Experiment (**A**) with fast dynamics. Right: Experiment (**B**) with slow dynamics. During the experiments, reconstructions are performed and visualized on three slices.

In this article, we explore the new concept, with the aim to enable real-time tomography with Deep Learning-based image processing in tomographic pipelines. For this purpose, we replay two experiments with the RECAST3D software, using data obtained from our FleX-ray laboratory at CWI, in Amsterdam^5^. The experiments entail tablets that are dissolved in a fluid inside a glass container with a granular ground (see Figs. 1 and 2). Experiment **(A)** features large spatial structures and fast dynamics, whereas Experiment **(B)** has smaller structures and slower dynamics. They are representative for the study to bubble physics, an important topic in material and engineering sciences^12^, as well as dissolution processes, which is relevant in pharmaceutical research^13^. Due to natural noise and artefacts, the experiments allow for a real-world challenge of our learning strategy. The article is organized as follows: First, in Methods, we formalize real-time tomography, and explain the Deep Learning context and self-supervised denoising task called Noise2Inverse^14^. We furthermore discuss our software set-up and software contributions. In the subsequent section on Just-in-time Learning, we introduce three topics that we identified as main challenges: the stochastic structure of real-time data, suitable network architectures, and an online learning strategy. In the penultimate section, Results, we report on the findings for each topic with DNNs trained on our experimental data. We finalize with a discussion of the potential and remaining challenges of just-in-time learning.

## Methods

### Real-time computed tomography

Tomographic reconstruction is an imaging technique that probes the interior of an object using a penetrating beam, such as X-rays or electrons. In real-time X-ray CT, radiographic projections ${\textbf{f}}~\in ~{\mathbb {R}}^{m_x \times m_y}$ are recorded in a continuous stream while the object is rotated. A reconstruction algorithm then takes the last $m_\phi$ projections from the stream, usually corresponding to a full rotation, and recovers the interior of the object as a three-dimensional image ${\textbf{x}}_t~\in ~{\mathbb {R}}^{n_x \times n_y \times n_z}$. The reconstruction algorithm achieves this by inverting the X-ray transform *F*. That is, it solves ${\textbf{x}}_t$ from the relation $[{\textbf{f}}_{t-m_\phi }, \dots , {\textbf{f}}_{t}] = F({\textbf{x}}_t)$, with *t* the index of the last projection. Two major classes of reconstruction algorithms are used in practice. *Direct* algorithms solve the inverse problem analytically, by formulating the solution in a closed-form equation. Iterative algorithms, on the other hand, express the inverse through an optimization objective. Commonly used direct algorithms are the filtered-backprojection (FBP) and Feldkamp-Davis-Kress (FDK)^15^, and consist of a single, fast filtering operation and a subsequent application of $F^T$, the adjoint of the X-ray transform. Iterative algorithms often have better noise-reducing properties than direct algorithms, but require multiple applications of *F* and $F^T$, which increases the computational cost and time considerably^16^.

A (Deep-Learned) image processing or analysis task, such as a denoising algorithm, takes a reconstructed volume ${\textbf{x}}_t$ as input, and produces an output ${\textbf{y}}_t$. The output can be an image, but can also be a different quantity, such as a vector field or scalar, depending on the image task. We will denote the analysis algorithm with the mapping $A :{\textbf{x}}_t \mapsto {\textbf{y}}_t$. In the forthcoming sections, we will explain RECAST3D, a pipeline for reconstruction and visualization, and choose an image-to-image Deep Learning component for *A*. In a real-time tomographic pipeline, the processes of *Experimentation* until *Visualization/automation*, i.e., the different components of Fig. 1, are taking place concurrently, using software buffers, in order to be efficient. As a result, multiple reconstructions or analysis outputs can be generated from the same projections at time *t*. For brevity, we will reuse the subscript *t* amongst ${\textbf{f}}_t$, ${\textbf{x}}_t$, and ${\textbf{y}}_t$.

### Experimental set-up with RECAST3D

**Figure 3:**
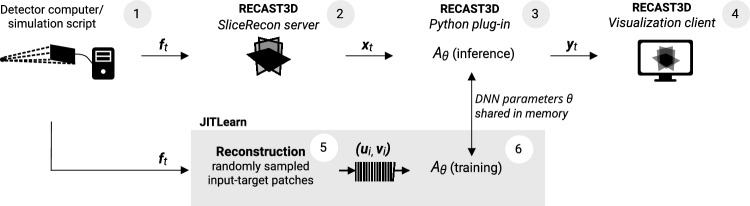
The proof-of-concept set-up for just-in-time learning with RECAST3D, using a U-Net for image denoising with the Noise2Inverse loss. Labels 1–4: The RECAST3D pipeline reconstruct slices ${\textbf{x}}_t$ from a stream of projections ${\textbf{f}}_t$, and sends these to the visualization client (cf. Fig. 2). Labels 5 and 6: Our *JITLearn* software intercepts the slices, via a plug-in, at (3). In a concurrent process, the U-Net architecture $A_\theta$ is trained on reconstructions $({\textbf{u}}_i, {\textbf{v}}_i)$, using a capacity queue (cf. Fig. 6). The figure is described in more detail in the main text of the section Software set-up.

High-resolution 3D image reconstruction poses a difficulty for real-time applications. To compute a full volume at the potential resolution of the data, algorithms take several minutes of computation time, even when modern hardware and efficient direct solvers are considered. To enable visualization and analysis at much faster frame rates, RECAST3D, a software for real-time reconstruction^6^, limits the reconstruction to a few user-selected slices through the volume. The RECAST3D graphical user interface is shown in Fig. 2. RECAST3D uses the FBP and FDK algorithms, which, thanks to their linearity and computational structure, allow reconstructing a region of interest with a computation time that is linearly related to the number of voxels in the region. The result is called a *quasi-3D* reconstruction, and permits a refresh-rate of milliseconds. In its graphical user interface, a user may add, remove, and reposition slices, which allows for a fast interrogation of the object during the experiment. RECAST3D has been used for real-time alignment^8^, explorative imaging^5^, and visualization of experiments with quickly evolving dynamics^9^. In our software configuration, we send the output of the analysis operator, ${\textbf{y}}_t$, to the graphical user interface, using RECAST3D’s package-based communication protocol. Reconstruction is therefore not restricted to slices, i.e.,  ${\textbf{x}}_t$ could also be a 3D image. The analysis operator, *A*, on the other hand, must always return a slice. By default, RECAST3D initializes with three orthogonal slices (Fig. 2). For the ease of discussion, however, we will limit our analysis to a single slice. The RECAST3D pipeline is illustrated in the top four processes of Fig. 3. We will discuss its technical details in the section Software set-up.

For our experiments, we take a subset of projection data from the two dynamic imaging experiments^5^, each corresponding to a five-minute interval. The laboratory set-up consists of a polychromatic conebeam source and a Dexela1512NDT CMOS detector. Measurement data is acquired by rotating the sample at a speed of 1.8 seconds per rotation. With an exposure time of 12 milliseconds per frame, this yields 150 projections for each full rotation. The field of view was cropped to the sample holder, which resulted in a 647-by-768 pixel image. Flat and dark field images were collected before the start of the experiment. For reconstructions, we used a voxel size of 0.25 mm and the geometry parameters that are provided with the data. Reconstructions are computed from full rotations (150 projections) with the FDK algorithm, which is a type of filtered-backprojection for the conebeam geometry. For spatio-temporal reconstructions, the interval between neighbouring reconstructions is fixed to 0.720 seconds (60 projections).

### Deep learning for real-time analysis

*Deep Learning* is a recent machine learning technology which obtains impressive results in various fields of imaging sciences, e.g., autonomous driving, robotics and computer vision, by using deep neural network architectures with convolutional layers and non-linear activation functions^17,18^. DNNs enjoy remarkably fast execution due to execution on GPUs. They are therefore especially well-suited in tomographic pipelines, where they can be used to perform real-time image enhancement tasks such as supersampling, artefact removal, segmentation, or denoising^11,19,20^. Fast inference is of crucial importance in synchrotron and laboratory environments, to yield real-time feedback at sufficient framerates as well as to create control mechanisms that steer the ongoing experiment. From here on, the analysis operator $A_\theta$ of Fig. 3 will denote an image-to-image DNN.

In general, the goal of *A* is to perform an image task by approximating an ideal mapping $A^{\dagger } : {\mathscr {U}} \rightarrow {\mathscr {V}}$, where ${\mathscr {U}}$ and ${\mathscr {V}}$ denote image manifolds. For image denoising, for example, the aim of *A* would be to approximate a perfect denoiser: Then $A^\dagger$ would map each noisy image from ${\mathscr {U}}$ to a noise-reduced counterpart in ${\mathscr {V}}$. The approximation of DNNs is accomplished through a parametrization of the mapping, i.e., $A = A_\theta$, with $\theta \in \Theta$ being the free parameters, such as weights of convolutional filters. Deep Learning concentrates on three main aspects: representation, optimization, and generalization. In the following, we describe each aspect in the context of real-time tomography, and introduce further notation.

Designing a DNN architecture $A_\theta$ that is well-suited to the task at hand, is the area of *representation*. A design usually concerns the number of layers and channels, choices of operators, and connections in the network. In the context of tomographic pipelines (Fig. 3), an architecture has the possibility to use multiple reconstructions as input, to take advantage of the spatio-temporal information contained in the data.

During *optimization*, or *training*, $\theta$ is optimized using a data set of input-target pairs. We will use ${\textbf{u}}_i \in {\mathscr {U}}$ to denote an input—a 2D or 3D reconstruction—and ${\textbf{v}}_i \in {\mathscr {V}}$ to denote a target. In *supervised learning*, the target is a ground truth, i.e., ${\textbf{v}}_i \approx A^\dagger ({\textbf{u}}_i)$. In the case of supervised denoising, for example, the input would be a noisy image, and the target is the noise-reduced counterpart. The inputs and targets do not need to correspond to the data seen during inference, that is, ${\textbf{u}}_i$ and ${\textbf{v}}_i$ can be different to ${\textbf{x}}_t$ and ${\textbf{y}}_t$. In our case, ${\textbf{u}}_i$ and ${\textbf{v}}_i$ are random small regions-of-interest reconstructions: Image-to-image networks can be designed to work for inputs of different dimensions, which, as we will see, speeds up the training process significantly. Given a task-specific *loss function* $\ell ({\textbf{u}}, {\textbf{v}})$ that measures the misfit between two images, training can be formulated as the optimization of the *empirical risk* $R_{{\mathscr {D}}}$ for parameters $\theta$, data set ${\mathscr {D}}$, architecture $A_\theta$ and loss $\ell$:

1 $$\begin{aligned} \min _{\theta \in \Theta } \quad R_{{\mathscr {D}}}[A_\theta ] := \frac{1}{|{\mathscr {D}}|} \sum _{({\textbf{u}}_i, {\textbf{v}}_i) \in {\mathscr {D}}} \ell \left( A_\theta ({\textbf{u}}_i), {\textbf{v}}_i\right) . \end{aligned}$$

The performance of a trained network on unseen data is the domain of *generalization*. To quantify generalization, image pairs from ${\mathscr {D}}$ are considered as random samples from a latent *training distribution*, a probability distribution ${\mathbb {P}}$ over ${\mathscr {U}} \times {\mathscr {V}}$. The *expected risk* describes the true but unknown performance of $A_{\theta }$ over ${\mathbb {P}}$,

2 $$\begin{aligned} R_{{\mathbb {P}}}[A_\theta ] := {\mathbb {E}}_{({\textbf{u}}, {\textbf{v}}) \sim {\mathbb {P}}} \left[ \ell (A_\theta ({\textbf{u}}), {\textbf{v}}) \right] , \end{aligned}$$

whereas the empirical risk is limited to a finite sample of it. The *generalization gap*, $\left| R_{{\mathscr {D}}} - R_{{\mathbb {P}}}\right|$, describes the distance between the two risks, and a network is said to generalize well when the gap is small. To quantify the gap, $R_{\mathbb {P}}$ is commonly approximated using a *hold-out data set* ${\mathscr {D}}'$ also sampled from ${\mathbb {P}}$ (also often referred to as the *test data set*), and one computes $\left| R_{{\mathscr {D}}} - R_{{\mathscr {D}}'}\right|$ for an approximation of the generalization gap. In our setting, ${\mathscr {D}}$ and ${\mathscr {D}}'$ contain reconstructions, and need to be generated from different projection sets to be independent samples from ${\mathbb {P}}$.

### Self-supervised image denoising with Noise2Inverse

An important analysis task for real-time tomography is image denoising. Reconstruction images are often severely degraded due to noise that the reconstruction algorithm propagates from the projection data. This is unavoidable in most real-time experiments, as low angular sampling and short exposure times are necessary to observe fast object dynamics. Unfortunately, fast direct reconstruction algorithms, including the filtered-backprojection, are less capable of suppressing noise. In the past years, several DNNs, e.g., DnCNN^21^, U-Net^22^, or the Mixed-scale Dense Network^23^, have demonstrated excellent denoising qualities. Moreover, denoisers can already be trained with small data sets, as the small-scale image features in a spatio-temporal neighbourhood of the target often provide sufficient information for the task. For example, the neural network approach named Noise2Self^24^ demonstrates that even a single image can be sufficient to obtain a noiseless output. Denoising is therefore an illustrative task for our just-in-time approach.

For denoising of tomographic data, different training strategies are used in practice. The supervised strategy is to train with a noiseless target. However, such strategy is not feasible in tomographic pipelines, as noiseless ground truths cannot be obtained in reasonable time. In such cases, the self-supervised learning methodology called *Noise2Noise*^25^ can be implemented, which allows DNNs to approximate the noiseless ground truth using image self-similarity. Noise2Noise requires a training data set ${\mathscr {D}}$ consisting of pairs $({{\textbf{u}}}_i, {{\textbf{v}}}_i)$, where ${{\textbf{u}}}_i$ and ${{\textbf{v}}}_i$ are images of the same object but contain different i.i.d. (independent and identically distributed) realizations of a latent noise distribution. The use of a noisy target, rather than a ground truth, yields an optimization problem where $A_\theta ({\textbf{u}}_i)$ needs to match ${{\textbf{v}}}_i$, polluted with a different noise realization than the input. However, since unbiased noise does not correlate between inputs and targets, $A_\theta$ can at best reproduce noiseless image features, as these are consistent between ${{\textbf{u}}}_i$ and ${{\textbf{v}}}_i$. Those image features are encoded as convolutional filters in $\theta$, and are learned due to similarity of patterns in the data set.

Noise2Noise is brought to the tomographic domain by *Noise2Inverse*^14^. Since ${{\textbf{u}}}_i$ and ${{\textbf{v}}}_i$ need to be generated from the same set of projections, $[{{\textbf{f}}}_{t-m_\phi }, \dots , {{\textbf{f}}}_t]$, Noise2Inverse proposes to split the projections into sets of odd and even time indices. When ${{\textbf{u}}}_i$ and ${{\textbf{v}}}_i$ are subsequently computed from the different sets, they result in closely similar objects, but with statistically independent noise. However, as fewer projections are used in reconstruction, the method sacrifices angular resolution.

### Software set-up

For the proposed just-in-time learning strategy, reconstruction software and Deep Learning software need to work closely together. In Fig. 3, we illustrate our proof-of-concept set-up. The arrows from (1) to (4) describe the RECAST3D pipeline, which is built on top of its *TomoPackets* library, i.e., a set of software interfaces for passing projections, geometries, reconstructions and user interactions via Push/Pull and Publish/Subscribe protocols in ZeroMQ^26^. Real-time reconstruction requires two processes: one to compute ${\textbf{x}}_t$ at fixed projection intervals in the RECAST3D *SliceRecon* server, component (2), and another to continuously generate training data $({\textbf{u}}_i, {\textbf{v}}_i)$, at (5). Deep Learning requires two similar processes. The first, a Python script at component (3), accepts ${\textbf{x}}_t$, loads $\theta$ from memory, and subsequently computes ${\textbf{y}}_t = A_\theta ({\textbf{x}}_t)$ and sends it to an external machine running RECAST3D. Component (6) accepts a batch of $({\textbf{u}}_i, {\textbf{v}}_i)$, performs a training step, and updates the stored parameters $\theta$. In an optimal set-up, the four processes run concurrently, each with a single or multiple GPUs.

In the proof-of-concept software, *JITLearn*, that we release together with this article (see Additional Information), we separate training, i.e., we only run component (5) and (6), and evaluate the trained network at a later time. To simulate a real-time experiment, we preload all projection data to RAM (*Random Access Memory*), and synchronize all GPU processes using a projected experiment start time. Projection data is subsequently released to the GPU processes on the basis of a virtual framerate, which we set to 24 milliseconds per frame to prolong our experimental data from 5 to 10 minutes. Dark and flat field removal, as well as FDK filtering steps, are not computed immediately, but processed on the GPU as soon as required by a reconstruction. During training, we store the network parameters at regular intervals. This allows restoring and evaluating the network on different ${\textbf{x}}_t$ afterwards.

The neural network architecture we employ for our experiments with Noise2Inverse is a U-Net^22^. This is a widely adopted architecture for image processing, consisting of a symmetric downscaling (encoder) and upscaling (decoder) part complemented by skip connections. In our PyTorch implementation, which replicates the original U-Net proposal^22^, downscaling is performed with strided convolutions, and upscaling with transposed convolutions. Our U-Net has implemented three down and upscaling levels. Each level has three encoding and three decoding convolutional layers, with a concatenating skip connection bridging the two parts. Every level doubles the number of feature maps while halving their resolution, starting with 8 feature maps after the first level. A higher number of levels increases the size of the network’s *receptive field*, i.e., the region in the input that, after a sequence of layers, contributes to the determination of a feature. Our implementation accommodates to reconstructions that are *2D*, *2D + time*, or *3D*. Both *2D + time* and *3D* are implemented with 3-dimensional convolutions, rather than a concatenation in the channel dimension. We thus treat a third dimension similar to the first two. We refer to this architecture without modifications as our *Standard* architecture.

For training, we employ the *Adam* optimizer with a learning rate of 0.0001 and batch size of 32. We do not train on full slices, but reconstruct only small regions-of-interest in the volume, which we refer to as *patches*. The patches are sampled uniformly at random from a slice as well as its spatial neighbourhood in the fluid container. No patches are sampled outside the glass container to prevent the sampling of reconstruction artefacts. While the patch-sampling region is adjustable during the experiment, we currently do not provide a graphical user interface to do so—by default, the optimizer samples from the entire reconstruction volume. For brevity, we will not introduce notation for this sampling process in our analysis or results.

### Training data generation

Generating sufficient amounts of reconstruction data poses a key challenge when training in parallel with the ongoing experiment (cf. component (5) in Fig. 3). A naive approach would call a reconstruction software for each ${\textbf{u}}_i$ and ${\textbf{v}}_i$, and with that repeat projection preprocessing steps, as well as CPU-GPU memory transfers, unnecessarily. Obtaining $({\textbf{u}}_i, {\textbf{v}}_i)$ as random samples from a single high-resolution volume is not possible either, as high-resolution 3D reconstruction takes seconds to minutes to complete^6^. This would result in either throttling the training process, or introducing a delay before the network can access new reconstruction data.

This problem has motivated the development of a new software package, which we released as an extension to the GPU-accelerated reconstruction framework called ASTRA Toolbox^27^. Our software takes the Nvidia CUDA kernels from the ASTRA package through *CuPy*, a Python interface to the Nvidia CUDA library. The software enabled an implementation of the FDK algorithm that is optimized for repeated reconstructions from a streaming buffer of projection data. Before entering the buffer, projections are pre-filtered with the Ram-Lak filter and stored in *textures*, a CUDA memory structure for efficient interpolation. Modifications to the CUDA kernels furthermore enable efficient backprojection from arbitrary projection subsets, which benefits the efficiency of the Noise2Inverse algorithm.

**Figure 4:**
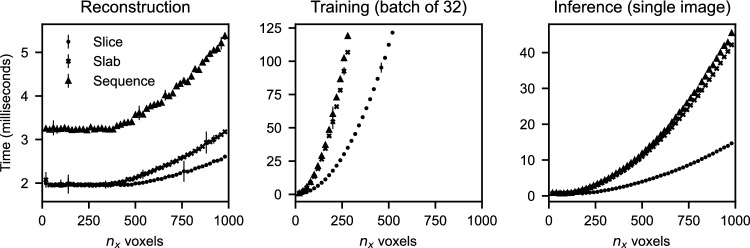
Timings, in milliseconds, for $n_x$-by-$n_x$ slices ($\bullet$), $n_x$-by-$n_x$-by-3 slabs ($\times$), and three consecutive slices ($\blacktriangle$). DNN timings use the *Standard* architecture. Training time includes backpropagation, whereas inference time only measures the forward pass. Timings are calculated using the mean over 200 repetitions after a 200 warm-up iterations on an Nvidia GeForce RTX 2080 Ti Rev. A.

Figure 4 shows run-times of the developed software for differently sized reconstructions. With *slices* we denote 2D reconstructions, with *slabs* thin reconstruction volumes, and with *sequences* three consecutive reconstructions. A small training patch takes about 2 milliseconds, regardless of size, as the computational cost of the reconstruction algorithm for very small reconstructions is dominated by geometry computations. Timings of DNNs for different input sizes display an expected quadratic trend in $n_x$, i.e., a linear relation to the total number of voxels. For training with small patches, reconstruction is the bottleneck. A batch of 60-by-60 reconstructions, for example, takes 64 milliseconds, while an iteration of the optimizer for these inputs takes 10 milliseconds. During inference, on the other hand, the DNN is typically the bottleneck. Inference on a 1000-by-1000 slab takes about 45 milliseconds (22 Hz), and reconstruction is an order of magnitude faster.

## Just-in-time learning

We will now sketch a context for the just-in-time learning paradigm by formulating three interlinked research topics. The first topic formalizes the continuously varying data that is encountered during an experiment. In the second topic, we discuss the DNN architecture and its relation to the reconstruction operator. In the third topic we explain the buffer-based online training approach that we developed for pipeline data. We will take the assumption that no previous training data, or ground truths, are available prior to the experiment.

### Topic I: real-time imaging data

In a real-time scientific imaging experiment, reconstructions are often originating from a continuous (physical) process. A data set ${\mathscr {D}}$ hence consists of consecutive reconstructions. The real-time setting is in that regard different to typical neural network applications, where the data set is often assumed to consist from i.i.d. (image) samples of a distribution ${\mathbb {P}}$ over ${\mathscr {U}} \times {\mathscr {V}}$. Instead, real-time samples are often highly temporally correlated, and describe a type of directed random walk on the manifold of ${\mathscr {U}} \times {\mathscr {V}}$. We will see that this is both a “curse and a blessing”: while these samples may not appropriately cover the sought data distribution, short sequences of correlated data contain local information that can help neural networks with the analysis task (discussed in Topic II).

A more formal modelling of real-time data in the tomographic pipeline would describe ${\mathscr {D}}$ as a single realization of a continuous stochastic process with the Markov property^28^. Every input-target pair $({\textbf{u}}, {\textbf{v}})$ from ${\mathscr {D}}$ can thus be interpreted as a sample from a time-dependent ${\mathbb {P}}_t$ over ${\mathscr {U}} \times {\mathscr {V}}$ that is correlated with all previous samples. The evolution of ${\mathbb {P}}_t$ reflects the evolution of the physical system that is imaged: If the system is in a (possibly dynamic) equilibrium, ${\mathbb {P}}_t$ remains constant over time (a *steady-state* regime). While subsequent samples are still temporally correlated, a long collection of samples will eventually approximate the distribution. In *transient* regimes, ${\mathbb {P}}_t$ changes smoothly with *t*, and each sample is from a slightly different distribution. Transient regimes occur at the beginning of an experiment, during experiments with evolving dynamics, or, for instance, during object exploration^5^ and zooming^4^. They pose a bigger challenge for learning. Obtaining sufficient samples to cover a particular ${\mathbb {P}}_t$ might require multiple realizations of the same process, i.e., repetitions of the experiment under the same conditions.

However, ${\mathbb {P}}_t$ may also change abruptly in time, e.g., if the user changes the scan geometry or alters a reconstruction parameter during the experiment. In RECAST3D, a re-orientation of a slice effectively changes the geometry of the reconstruction, and the images may show differently-oriented features of the object. We call these *discontinuous jumps* between continuous regimes. Draws from ${\mathbb {P}}_t$ at a time *t* and its next timestep, at $t+1$, could possibly display an unseen part of an object, and therefore radically change the latent distribution of the data samples. A network trained on a data set ${\mathscr {D}}$, with samples drawn until ${\mathbb {P}}_t$ at time *t*, is thus principally unprepared to generalize to samples after a jump, say a data set ${\mathscr {E}}$ drawn at time $t+1$, as they are not from the training distribution. In Results I, we therefore quantify the discontinuous jumps, and in Results III, we investigate the ability of a network to recover from a discontinuity.

### Topic II: network architectures

In order to function in a just-in-time setting, DNN architectures need to be comparatively small. That is, they need to consist of a limited number of (parameterized) operations, such as layers and channels. The first reason is that, in order to keep up with the incoming data from the tomographic pipeline, the network optimizer must be able to update network parameters quickly enough, which is only possible when the number of operations is small. The second reason is that, during inference, a network is presented images in high resolution. For large detectors, for example, a reconstructed slice at the potential resolution of the data can be as large as 2000-by-2000 pixels. Large networks typically demand too much GPU memory and cannot easily attain high framerates at these resolutions. On the other hand, small networks have limited expressivity, which makes a careful architectural design (e.g., number of hidden layers, residual connections) more important. For image tasks with low spatio-temporal complexity, such as segmentation and denoising, small networks yield satisfactory results^20^.

**Figure 5:**
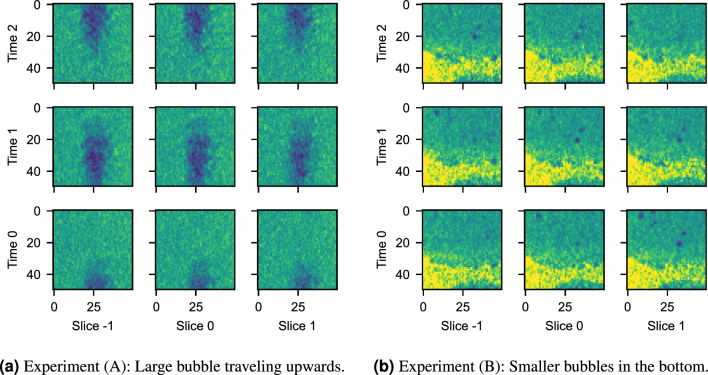
Different dynamics between Experiments (**A**) and (**B**), illustrated by the spatio-temporal reconstruction of a vertically-oriented subvolume of dimensions (50, 50, 3). In (**a**), the dissolvent is soap-based, leading to fast-traveling bubbles of overall large size. In (**b**), a spatio-temporal pattern appears along the time-slice diagonal, due to the slower and smaller bubbles in the gel-based dissolvent.

The next consideration for an architecture is what additional reconstructions it should use as inputs to process a given image slice. Neighbouring slices and additional timesteps—usually given to the network as additional image channels—often provide important contextual information, which could help to improve the image task. Fig. 5 shows this by example, where, in a region of interest, Experiment **(A)** has spatially coherent features (along rows), while Experiment **(B)** has spatio-temporally coherent features (along diagonals). For a denoising task, where redundant patterns contain additional information, a network for Experiment **(A)** may benefit from a sequence of nearby slices, whereas a network for Experiment **(B)** improves with a spatio-temporal volume. More generally, this also points to an intricate connection between reconstruction and analysis operators (*G* and *A*). Multiple spatial or temporal reconstructions may improve the network performance, but also decrease the reconstruction speed, and delay training and the end-to-end framerate of the pipeline. A network should therefore ideally be designed jointly with all parameters of the reconstruction operator, such as the spatial extent, the choice of filter, or image resolution. We will illustrate the importance of architectural choices in Results II.

### Topic III: online learning

*Online learning*, or incremental learning, is a strategy in which samples are presented in sequential or chronological order during training^29^. The strategy is typically employed in machine learning applications where data is processed in large volumes, as it allows data samples to be discarded after training. During online learning, network weights are continuously updated to fit the most recently presented samples. As a result, a network that generalizes well to a distribution of recent samples may have diminishing accuracy for older samples—a process known as *catastrophic forgetting*. While this behaviour is undesired for learning a static distribution from a stream of data^30^, the strategy suits real-time tomography, since distributions are time-dependent and our goal is to generalize only to the most recently reconstructed image from the pipeline. The field of online learning as of today is still developing, but examples exist in image classification^31^ and denoising autoencoders^32^.

*Just-in-time learning* is an application of real-time online learning to dynamic imaging data (Topic I), and can be explained through the temporal evolution of neural network weights $\theta$. Without the real-time aspect, its goal at a time *t* is to find optimal weights $\theta ^\star _t$ from reconstructions ${\mathscr {D}}_t$ (Eq. 1) obtained by drawing from ${\mathbb {P}}_t$. Note that, since pairs $({\textbf{u}}_i, {\textbf{v}}_i)$ are generated separately from ${\textbf{x}}_t$ (Fig. 3), such ${\mathscr {D}}_t$ would ideally be designed *ad hoc* by reconstructing input-target pairs that are expected to help generalization towards ${\textbf{x}}_t$. For example, when ${\textbf{x}}_t$ is from a *steady-state* regime, ${\mathscr {D}}_t$ could consist of a large amount of reconstructions sampled from the last *m* projections $\{{\textbf{f}}_{t-m}, \dots , {\textbf{f}}_t\}$ corresponding to the regime. As each time index has a corresponding data set and optimum, the dynamics of the real-time experiment leads to a trajectory $\theta ^\star _0, \theta ^\star _1, \dots$ in the parameter space, and in principle we would like our learning process to follow this trajectory as closely as possible.

In practice, the challenge of just-in-time learning is not only to approximate $\theta ^\star _t$ at each time *t* as closely as possible, but also to do this within the time constraints of the experiment. Similar to other online strategies, we achieve this by continuing the training with the previous (suboptimal) weights $\theta _{t-1}$ and by appending or updating the data set ${\mathscr {D}}_{t-1}$ with new samples to form ${\mathscr {D}}_{t}$. Thanks to the spatio-temporal continuity of ${\mathbb {P}}_t$, the difference between most data set pairs is small, and an optimization from $\theta _{t-1}$ towards $\theta ^\star _{t}$ can therefore be interpreted as a minimal example of *transfer learning* from ${\mathscr {D}}_{t-1}$ to ${\mathscr {D}}_{t}$. Transfer learning can be significantly faster and a neural network can reuse features as well as low-level statistics when $\theta _{t-1}$ is in the *basin* of $\theta ^\star _{t}$^33^. A basin is a region from the parameter space in which local descend optimization methods, e.g., the commonly-used stochastic gradient descend, would converge towards $\theta ^\star _{t}$. Our approach is different to, e.g., *continual learning* where instead the goal is to preserve the structure of the parameter space, for example through constraints on the weights or gradients, or by replaying training data^34^.

**Figure 6:**
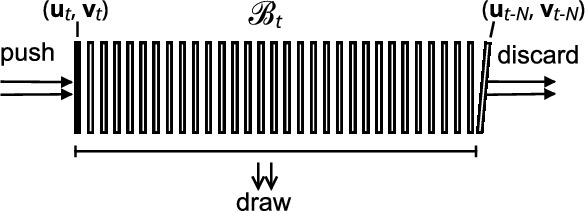
Online learning implemented with an $N_{\text {buffer}}$-sized capacity queue ${\mathscr {B}}_t$. Training-target pairs $({\textbf{u}}_t, {\textbf{v}}_t)$ are pushed onto the queue, while the network optimizer draws pairs asynchronously by a uniform random sampling of their indices.

In our result section, we train DNNs with reconstructions that are generated chronologically. However, as illustrated in Fig. 4, sample generation is often slower than optimization, even with our tailored reconstruction software. This forms a bottleneck: If we would allow only single-time access per sample, i.e., let ${\mathscr {D}}_t = \{({\textbf{u}}_t, {\textbf{v}}_t)\}$, in the way other online learning strategies are designed, the optimizer would not be able to continue training until new reconstructions are generated. We therefore introduce a modification that enables resampling of reconstructions for a short duration. We will let the training data set be time-dependent, i.e., ${\mathscr {D}} = {\mathscr {B}}_t$, and practically implement this as a buffering capacity queue, as shown in Fig. 6. The buffer ${\mathscr {B}}_t$ at time *t* contains the last $N_{\text {buffer}} := |{\mathscr {B}}_t|$ draws from ${\mathbb {P}}_{t}$. By enabling asynchronous drawing from the buffer, the optimizer may select randomly from ${\mathscr {B}}_t$ while new reconstructions are continuously added.

The size of the buffer, $N_\text {buffer}$, is a user-specified parameter. If an optimizer is able to handle $n_{\text {opt}}$ samples per second, and reconstructions are generated with $n_{\text {reco}}$ samples per second, a sample will on average be picked $n_\text {opt} / n_\text {reco}$ times during its lifetime in ${\mathscr {B}}_t$. The choice of $N_{\text {buffer}}$ thus does not affect how often a sample is picked. A suitable choice, however, may not be straightforward. A too small buffer may not contain sufficiently diverse features. In contrast, a buffer too large may unnecessarily retain reconstructions that have become irrelevant for the current timestep. In Results III, we will inspect the online learning process for different buffer strategies.

## Results

We investigate the three topics of the preceding section with the Noise2Inverse method as learning task and data collected using our experiments. To quantify results for samples in small time windows, the mean-squared error loss does not provide a robust performance metric, due to the fluctuating noise levels that occur during data collection in our set-up. Similar to the loss and expected risk (equation (2)), we therefore define an *accuracy* $\alpha$ and *empirical accuracy* $R^\alpha _{{\mathscr {D}}}$, with

3 $$\begin{aligned} \alpha (A_\theta , {\textbf{u}}, {\textbf{v}}) := \frac{\Vert A_\theta ({\textbf{u}}) - {\textbf{v}}\Vert _2^2}{\Vert {\textbf{u}} - {\textbf{v}}\Vert _2^2}, \quad R_{{\mathscr {D}}}^\alpha \left[ A_\theta \right] := \frac{1}{|{\mathscr {D}}|}\sum _{({\textbf{u}}_i, {\textbf{v}}_i) \in {\mathscr {D}}} \alpha \left( A_\theta , {\textbf{u}}_i, {\textbf{v}}_i\right) . \end{aligned}$$

In comparison to the mean-squared error (MSE), the denominator of $\alpha (A_\theta , {\textbf{u}}, {\textbf{v}})$ in Equation (3) additionally normalizes by the noise magnitude. As a result, we expect the empirical accuracy to be $R_{\mathscr {D}}^\alpha \in [0.5, 1]$. To see this, first consider a network that does not denoise at all, i.e., $A_\theta ({\textbf{u}}) = {\textbf{u}}$, and note that this leads to $\alpha (A_\theta , {\textbf{u}}, {\textbf{v}}) = 1$ and $R_{\mathscr {D}}^\alpha = 1$. Then, consider the ideal Noise2Noise setting (discussed in Methods) in which ${\textbf{u}} = \hat{{\textbf{u}}} + \epsilon$ and ${\textbf{v}} = \hat{{\textbf{u}}} + \epsilon '$, where $\hat{{\textbf{u}}}$ is the noiseless image and $\epsilon$ and $\epsilon '$ denote two independent realizations of voxelwise-i.i.d. noise images with zero mean and finite variance $\sigma ^2$. A perfect denoising network would predict $A_\theta ({\textbf{u}}) = \hat{{\textbf{u}}}$, and therefore

4 $$\begin{aligned} \alpha (A_\theta , {\textbf{u}}, {\textbf{v}}) = \frac{\Vert \hat{{\textbf{u}}} - (\hat{{\textbf{u}}} + \epsilon ')\Vert _2^2}{\Vert (\hat{{\textbf{u}}} + \epsilon ) - (\hat{{\textbf{u}}} + \epsilon ')\Vert _2^2} = \frac{\tfrac{1}{n} \sum _i^n \epsilon '^2_i}{\tfrac{1}{n} \sum _i^n (\epsilon _i - \epsilon '_i)^2}, \end{aligned}$$

where $n := n_x n_y n_z$ is the number of voxels. For large *n*, i.e., for typical image sizes, the nominator will be close to $\sigma ^2$ whereas the denominator will be close to $2\sigma ^2$, thus $\alpha (A_\theta , {\textbf{u}}, {\textbf{v}}) \approx 0.5$. Using the Noise2Inverse setting, however, we will occasionally see that $R_{\mathscr {D}}^\alpha < 0.5$. This is possible when sparse-angle artefacts or fast object motion inflicts additional differences between the even-angle (${\textbf{u}}_t$) and odd-angle (${\textbf{v}}_t$) reconstructions, in other words, when ${\textbf{u}}_t$ and ${\textbf{v}}_t$ have different underlying ground truths. When a DNN network learns to predict such differences, this reduces the numerator $\Vert A_\theta ({\textbf{u}}) - {\textbf{v}}\Vert _2^2$ in Eq. (3).

### Results I: real-time imaging data

As discussed in Topic I, a real-time network continuously encounters differently distributed samples, due to transient and discontinuous changes in the data distribution. We call these *out-of-distribution samples*. Compared to the hold-out samples, both types of samples have not been seen by the network before, but the out-of-distribution samples are not distributed like the training set. To quantify the error this induces, we train the *Standard* network $A_\theta$ on a data set ${\mathscr {D}}$, while evaluating it on samples of an unseen data set ${\mathscr {E}}$. At the same time, we also train a baseline network $A_\psi$ on ${\mathscr {E}}$. The accuracy change is approximated by

5 $$\begin{aligned} \epsilon := R^\alpha _{{\mathscr {E}}}\left[ A_{\theta }\right] - R^\alpha _{{\mathscr {E}}}\left[ A_{\psi }\right] \text {,} \end{aligned}$$

i.e., the difference between $A_\theta$, trained on ${\mathscr {D}}$ but evaluated on ${\mathscr {E}}$, and the baseline $A_\psi$, trained on ${\mathscr {E}}$ and evaluated on ${\mathscr {E}}$.

**Figure 7:**
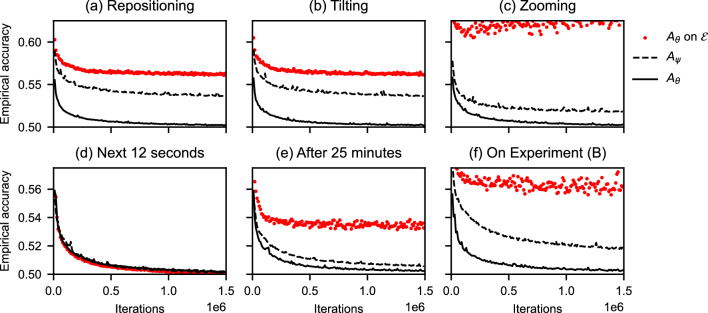
Empirical accuracy of a network with parameters $\theta$ trained on ${\mathscr {D}}$, and a network with parameters $\psi$ trained on ${\mathscr {E}}$. In (**a**)–(**c**), ${\mathscr {E}}$ is a change to ${\mathscr {D}}$ due to interaction with the experiment, and in (**d**)–(**f**), ${\mathscr {E}}$ is a data set from different projections. The solid and dashed lines denote the accuracies of $A_\theta$ and $A_\psi$ on 3200 hold-out samples. The error of $A_\theta$ on out-of-distribution samples, i.e., Eq. (5), is the distance between the potential accuracy, i.e., the dashed baseline $R_{{\mathscr {E}}}^\alpha [A_\psi ]$, and the achieved accuracy, i.e., the red markers $R^\alpha _{{\mathscr {E}}}[A_\theta ]$.

Figure 7 displays six scenarios for ${\mathscr {D}}$ and ${\mathscr {E}}$. Scenarios (a)–(c) correspond to discontinuous changes due to user interaction, and (d)–(e) show the transient regimes during the experiment. Both ${\mathscr {D}}$ and ${\mathscr {E}}$ are generated from 12 seconds of projection data of Experiment **(A)**. The error $\epsilon$ is the difference between the dashed line and the red dotted markers. For *repositioning*, ${\mathscr {D}}$ is the top half of the volume, and ${\mathscr {E}}$ the bottom half. For *tilting*, we change horizontal to vertical slices, and for *zooming* we reduce the voxel size to 0.125. In the second row of the figure, the choices for ${\mathscr {E}}$ are data sets that are generated from different projections. In (d), projections are taken 12 seconds directly after ${\mathscr {D}}$. In (e), the data is from 25 minutes later in the same experiment. For (f), 12 seconds of data are taken from Experiment **(B)**.

In Fig. 7 most scenarios show that evaluation on out-of-distribution samples cause a significant loss in accuracy, and that training $A_\theta$ for a prolonged time does not improve this evaluation. For user interaction, the error $\epsilon$ is highest in the case of *zooming*. This is expected, as convolutional operators are not scale-invariant, and therefore take notion of the size of the learned image features. We note that a similar change in the data distribution could also occur when the acquisition geometry changes. In panel (d), training on ${\mathscr {D}}$ generalizes well to the next 12 seconds. In (e), however, the object dynamics in Experiment **(A)** have changed substantially, as more smaller bubbles occur in the data. This led to a decreased accuracy. In (f), we find that generalization towards Experiment **(B)** is poor, likely due to the change in liquids and set-up. We will inspect the visual effect of out-of-distribution evaluation in Results III.

Our results in this section, for Noise2Inverse and Experiment **(A)**, indicate that small DNNs can be sensitive to changes in the pipeline data, i.e., the learned image features from 12 seconds of data are not generic enough to be useful for differently placed, oriented or zoomed slices. While this may suggest extending the training data ${\mathscr {D}}$ with reconstructions from ${\mathscr {E}}$, similar to data augmentation, this is not straightforward. Set-up changes or discontinuous changes in the physics cannot easily be anticipated by augmentation. Moreover, small networks may not be expressive enough to anticipate large amounts of data, and the generation of additional data will require more computational resources.

### Results II: network architectures

In the following, we explore the performance of different U-Net architectures, and look at the effect of reconstruction dimensions for Experiments **(A)** and **(B)**. Table 1 lists four variations of our *Standard* architecture, the network trained on randomly-selected 20-by-20 input patches. *Cx*-networks use *x* feature maps after the first layer (and then follow the U-Net principle). With more feature maps the *representational capacity* of the network increases, i.e., the network stores a larger number of filters. The next class, *Vx*-networks, use larger *x*-by-*x*-sized patches during training, which increases the receptive field during training. *Zx*-networks use *x* nearby spatial reconstructed slices, and *Tx*-networks use *x* timeframes, with the middle frame as target. Both increase the image input during training and inference, and use 3D convolutions. During 30 minutes of training, samples are continuously generated from a fixed 12-seconds data set of projection data, and previous reconstructions are allowed to be resampled by the optimizer to avoid throttling. This is representative of online training in an experiment where the data has no temporal component yet. For evaluation, 3200 hold-out samples are taken from a 12 second interval later in the experiment.

**Figure 8:**
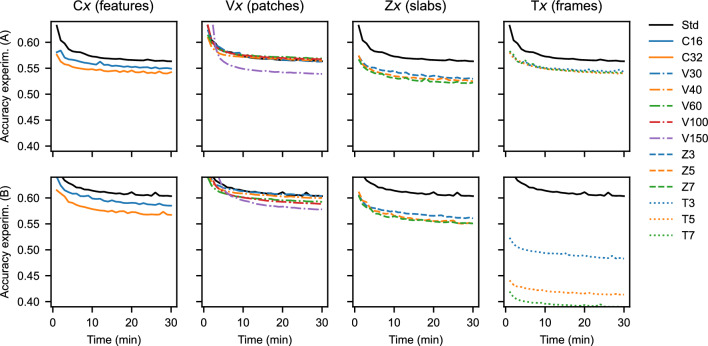
Empirical accuracy of the architectures in Table 1 for Experiment (**A**) and (**B**), using 3200 hold-out samples per evaluation point. Increasing the features (C*x*) or slices (Z*x*) is effective, although they slow down the DNN framerate. The effect of patch size (V*x*) and frames (T*x*) is dataset-dependent.

Figure 8 displays the evolution of empirical accuracies for the architectures presented in Table 1. The results show that all denoising networks can be trained to a satisfactory accuracy in 10 minutes or less. Training is approximately fast for all networks, although the *Cx*, *Zx*, and *Tx* networks become significantly slower when evaluated on high-resolution slices. This results in a lower framerate at the RECAST3D graphical interface. This is not the case for the *V30*–*V150* architectures, which used larger patches. While larger patches do not affect the DNN framerate, we note that the slower initial convergence was only valuable for Experiment **(B)**, where it eventually resulted in a better accuracy.

**Figure 9:**
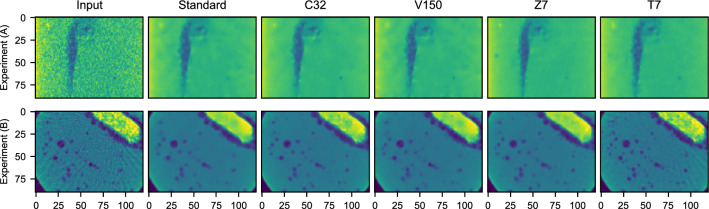
90-by-120 region-of-interest network outputs, comparing the best-performing DNNs in each class of Table 1 visually. *Standard*, C32, V150, Z7, and T7 outputs, for Experiment (**A**) and (**B**), are the best networks from Fig. 8 evaluated on *Input*. Experiment (**A**) shows a rising bubble in a vertically-oriented slice, and Experiment (**B**) a horizontal cross-section in the middle of the volume.

Figure 9 illustrates the best architectures in each category, with an evaluation on a hold-out sample. Overall, the architectures produce comparable outputs, suggesting that convergence speed may be the most important criterion for selecting a network. In Experiment **(A)** the more expressive network (*C32*) and more slices (*Z7*) help to produce a slightly smoother background. In Experiment **(B)**, *Z7* predicts bubbles better, and *T7* produces the sharpest bubble interfaces, although the latter predicted streak artifacts too. This is likely due to a difference that occurs between even-angle and odd-angle reconstructions in Noise2Inverse. Yet, since the dynamics in Experiment **(B)** are slower, the temporal network may also be better at using the redundancy of features between multiple frames.

**Table 1:** The network architectures in Topic II, corresponding to various parameter choices, that are used to compare accuracy and training speed in Figs. 8 and 9.

	Feature maps	Input dimensions	Frames	$n_\text {opt}$ (per second)	DNN framerate^*^ (Hz)
*Standard*	8	(20, 20, 1)	1	4,190	73
C16	16	$\cdot$	$\cdot$	4119	38
C32	32	$\cdot$	$\cdot$	4058	16
V30	$\cdot$	(30, 30, 1)	$\cdot$	3725	72
V40	$\cdot$	(40, 40, 1)	$\cdot$	4111	72
V60	$\cdot$	(60, 60, 1)	$\cdot$	2983	71
V100	$\cdot$	(100, 100, 1)	$\cdot$	1303	71
V150	$\cdot$	(150, 150, 1)	$\cdot$	591	71
Z3	$\cdot$	(20, 20, 3)	$\cdot$	3721	29
Z5	$\cdot$	(20, 20, 5)	$\cdot$	3820	17
Z7	$\cdot$	(20, 20, 7)	$\cdot$	3778	10
T3	$\cdot$	$\cdot$	3	4597	23
T5	$\cdot$	$\cdot$	5	4342	14
T7	$\cdot$	$\cdot$	7	3935	10

“$\cdot$” denotes a *Standard* parameter (see Software set-up). $n_\text {opt}$ denotes the number of samples per second drawn by the network optimizer during training. ^*^Corresponding to a 1024-by-1024 output slice.

### Results III: online learning

We conclude with a real-time simulation of Experiment **(A)** by replaying the full projection data set to our just-in-time software using the *T3* network (Table 1). When $t<336$ seconds, reconstructions are taken in random orientations from the top of the fluid container, and when $t\ge 336$ seconds, samples are taken from the bottom. This simulates a discontinuity in the pipeline data, corresponding to the repositioning of a horizontal slice in RECAST3D (panel (a) in Fig. 7). In this scenario, the reconstructions were generated at a speed of 9 batches per second, while the *T3* network trained with 141 iterations per second.

**Figure 10:**
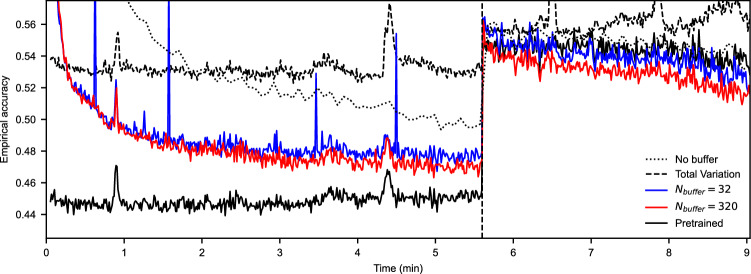
Accuracy of the online-trained *T3* network on Experiment (**A**), with different buffer sizes, in comparison to a pretrained baseline, Total Variation based denoising, and a training strategy without buffer. After 336 seconds, reconstructions are sampled from the bottom of the fluid container to simulate a discontinuity in the pipeline data. Each point on the graph is an evaluation of the network on 3200 randomly selected samples.

Figure 10 shows the empirical accuracy of the network during training, estimated with 3200 samples in a small window $\Delta t$ around each *t*, i.e., the accuracy $R^\alpha _{{\mathscr {D}}_{\Delta t}}$. The solid black line marks the accuracy of the *T3* offline-trained network on all data before the discontinuity, the dashed line a Total Variation (TV) based denoising^35^ implementation from the Python scikit-image package, and the dotted line describes a training strategy without buffer. The blue and red lines mark the just-in-time strategy with a buffer of 32 and 320 reconstructions, respectively. Our first observation is that both buffer sizes perform better than a network that processes the input sequentially. The network with $N_\text {buffer}=320$ (about a second of data), performs best, which suggests that including previous reconstructions in the training process is advantageous in the case of slow data generation.

**Figure 11:**
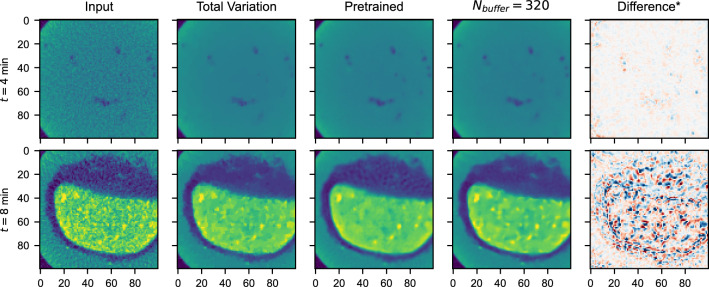
A comparison between the input image to denoise, denoising results of TV minimization, and pretrained and just-in-time trained network after $t=4$ and $t=8$ minutes, i.e., before and after the discontinuity of the experiment in Fig. 10. ^*^The difference is taken between the pretrained and $N_\text {buffer}=320$ outputs.

Figure 11 visualizes images of the pretrained and $N_\text {buffer} = 320$ network before and after the discontinuity. We note that at $t=4$ minutes (first row), bubbles of the just-in-time network are slightly oversmoothed, compared to the pretrained network, but that the results are of satisfactory quality. For TV, the regularization parameter was set to $\lambda =0.03$ and the number of iterations to $n_\text {iter}=300$, after tuning on 3200 samples from the top of the container. In these experiments TV denoising was not able to outperform the data-driven denoisers. In future work, classical TV can be combined with neural networks in the tomographic pipeline.

When, after 336 seconds, samples are taken from the bottom of the volume (see the tablet in Fig. 2), the pretrained and just-in-time strategies observe a similar accuracy. Interestingly, the just-in-time strategy requires only a short time to improve over the pretrained network. The second row in Fig. 11 shows, the strategy at $t=8$ minutes, has a clear visual advantage. It maintains the fine-grained structure in the tablet (yellow), and is better able to remove the noisy background in the gas around the tablet (dark blue). A video, combining Figs. 10 and 11 for the $N_\text {buffer}=320$ network, is included with the Supplementary Information.

## Discussion

Recent advances in Deep Learning have produced powerful image processing and analysis algorithms that operate in a fraction of the time that conventional algorithms take. Our *just-in-time* concept brings these algorithms to real-time tomographic pipelines at synchrotron light source facilities and X-ray microscopy laboratories, where a large diversity of imaging data is processed. The leading principle of just-in-time learning is that continuous regimes in the experiment lead to spatio-temporal continuous data in the pipeline, which can be be used to train while the experiment is ongoing. By combining small, easy-to-train architectures with online learning, the approach allows the analysis of reconstructions during the experiment, and can therefore be employed without preparation and without prior data. In this article, we have demonstrated just-in-time learning using the quasi-3D reconstruction paradigm proposed in RECAST3D^6^, and for the small U-Net architectures^20,22^ on the Noise2Inverse loss^14^. For extending it to high-resolution fully-3D reconstructions, fast DNN inference poses the main challenge. While image partitioning techniques can divide the workload over multiple GPUs^36,37^, such techniques have not yet been demonstrated on millisecond timescales.

In Results I, small networks are found to be sensitive to changes in the data distribution, even within a single experiment. This is generally unavoidable, since, as Results II illustrates, more expressive networks are significantly slower. At the same time, Results I and II show that reconstructions have a large degree of spatio-temporal continuity when the experiment is in steady-state or transient regimes. This enables spatio-temporal DNNs, and allows a neural network to generalize to data from the tomographic pipeline. In Results II, several hyperparameter settings, such as the number of feature maps, led to good visual results. However, we did not discuss how such hyperparameters are to be picked for a new set-up, task, or experiment. An interesting direction for future work would be to find heuristics to automate this. For Results III, we showed a straightforward buffer strategy for online learning. Yet, it is also possible to design the buffer or training data set adaptively. Constraints on gradients, or specialized network architectures, are further possibilities to retain information over longer time spans of the experiment^34^. This, and a more precise description and exploration of the formal concepts behind just-in-time learning that we sketched in Topic I and III, are important directions of future research.

We have demonstrated the feasibility of our approach using real-world laboratory data on an image denoising task using the Noise2Inverse training loss. While we expect this to be representative of other tasks with low spatio-temporal complexity, such as image super-resolution, segmentation, or artefact removal, future research is needed to see what is achievable for more sophisticated image processing tasks—especially when more diverse image features need to be stored in the network. Strategies such as dynamic pruning^38,39^, improved (U-Net) architectures^40^, mixed precision weights, and parallelization can improve the speed and accuracy of DNNs, which would widen the applicability of our approach by enabling more expressive networks. For denoising, *just-in-time* learning already proves to be a viable Deep Learning paradigm with a large potential use. Deep Learning-based analysis can provide valuable feedback during an experiment, which can be used for experimental control^8^ or real-time scan adaptation and optimization techniques. For the latter, we will investigate coupling the proposed just-in-time learning strategy with reinforcement learning approaches^41^.

### Supplementary Information

Supplementary Information.

## Data Availability

A subset of 10,000 projections of experimental data has been published on Zenodo^42^ at https://zenodo.org/records/3610187. The full data sets used in the manuscript are available upon reasonable request. Requests should be addressed to Felix Lucka.

## Code availability

Our Python software, JITLearn, is available at https://github.com/adriaangraas/jitlearn. Reprints and permissions information is available at www.nature.com/reprints.
